# Improving Population Pharmacokinetic Modelling with Artificial Patients using Generative Artificial Intelligence

**DOI:** 10.1002/prp2.70241

**Published:** 2026-04-03

**Authors:** Verena Schöning, Felix Hammann

**Affiliations:** ^1^ Clinical Pharmacology and Toxicology, Department of General Internal Medicine, Inselspital, Bern University Hospital University of Bern Switzerland

**Keywords:** data set augmentation, ordinary differential equations, population pharmacokinetics, Wasserstein generative adversary networks

## Abstract

In population pharmacokinetics (PopPK), non‐linear mixed effects (NLME) models are used to simultaneously describe a drug's pharmacokinetics (PK) and dynamics (PD) in a patient population using systems of ordinary differential equations. In this field, machine learning is mainly used for data preparation, hypothesis generation, predictive modeling, and model validation. Some approaches to integrate artificially generated information have already been explored, but real‐world application is still limited. We therefore conducted a proof‐of‐concept study to analyze the ability of generative artificial intelligence (AI) to create artificial patient profiles to augment PopPK data sets and assess their influence on parameter estimates. We defined the pharmacokinetic parameters of a hypothetical drug and simulated the concentration curves of 20 patients. We then trained Wasserstein Generative Adversarial Networks (WGANs) with gradient penalty (GP) to generate artificial patients. The data distribution of original and artificial patients was statistically indistinguishable as shown by the Maximum Mean Discrepancy test. Therefore, the WGAN‐GP is neither overfitted, that is producing only single instances of artificial patients, nor underfitted, that is producing unrealistic artificial patients. We then combined different shares of original and artificial patients in separate data sets to build and compare PopPK model estimates. Addition of artificial patients led to narrower confidence intervals, indicating more robust parameter estimates, and accentuated the allometric effect of weight on the volume of distribution. In conclusion, we provide a proof‐of‐concept that generative AI can be used to augment pharmacokinetic data sets, with preliminary evidence suggesting improved parameter estimation.

## 1 Introduction

The use of population pharmacokinetics (PopPK) during drug development is recommended to identify differences in drug safety and efficacy among population subgroups [1]. One commonly applied technique is non‐linear mixed effects (NLME) modeling. Pharmacokinetics (PK) and dynamics (PD) data from patients within a population are described simultaneously in a system of ordinary differential equations (ODEs), while also accounting for inter‐ and intra‐subject variability and residual error by estimating subject‐specific random effects (common covariates include sex, weight, or renal function). With PopPK models, it is possible to determine the right dosage and dosing schedule in a personalized manner, both a priori and a posteriori. With the introduction of electronic health records (EHRs), the amount of available data increased significantly, particularly the number of potentially informative covariates. Machine‐learning (ML) algorithms are uniquely suited to handle large amounts of data and thus further advance pharmacometric modeling. Currently, ML is mainly used in data preparation, hypothesis generation, predictive modeling, and model validation [2]. Generative AI has received growing attention. These models can create new instances, such as artificial patients, by learning from existing data distributions. Initial studies using generative AI in pharmacometrics have already been conducted. Jiang et al. [3] compared three different generative algorithms (multi‐layer perceptron conditioning generative adversarial neural network (MLP cGAN), time‐series generative adversarial networks (TimeGAN), and probabilistic autoregressive (PAR) models) to generate synthetic pharmacokinetic/pharmacodynamics (PK/PD) data, as time‐series. The data set consisted of clinical design (time, dosing), PK/PD measurements, and static and time‐varying covariates (age, weight, sex). Janssen et al. [4] used generative models to impute missing data and simulate virtual patients. The first model focused on the generation of covariates using different probabilistic ML models based on directed acyclic graphs (DAG). The second model fitted neural spline models to learn the data set–specific joint distribution of patient age, weight, height, and Von‐Willebrand Factor levels. Nikolopoulos et al. [5] used Wasserstein generative adversarial networks (WGANs) to increase sample size within clinical studies by adding virtual patients. They showed that WGANs effectively augment sample size, reduce the risk of type II errors, and potentially decrease time, cost, and ethical concerns.

In this study, we aim to explore the use of generative AI in pharmacometrics by augmenting PopPK models with artificial patients. Our goal is to increase the population size and assess the influence on model parameter estimations. The artificial patients are expected to differ with respect to covariate combinations (e.g., height, weight) from those included in the original data set. We hypothesize that a PopPK analysis based on artificial and real patients can more concisely describe the observed data and may provide increased robustness, assessed as reduced variability of point estimates.

## 2 Material and Methods

### 2.1 Wasserstein Generative Adversarial Networks (WGAN)

A powerful ML algorithm in this field of generative AI is generative adversarial networks (GANs), which generate new data using deep neural network (DNN) learning techniques. The GAN is provided with original samples, and it then learns the underlying probability distribution. Properly trained, the GAN is able to generate additional examples matching the observed probability distribution [6, 7]. This technique is so capable and versatile that it can generate artificial human faces that are almost indistinguishable from actual human faces [8]. Despite this, a common failure mode of this algorithm results in the generation of only a single or few different outputs. This is referred to as ‘mode collapse’ and is undesirable, as the generated samples do not represent the entire original feature space.

**FIGURE 1:**
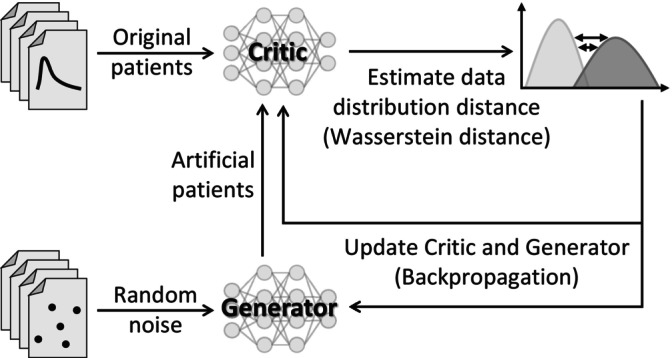
Structure of WGAN‐GP.

Wasserstein GANs (WGAN) [9] with gradient penalty (GP) [10] are less susceptible to this phenomenon. A WGAN‐GP consists of two competing DNN sub‐models: a generator and a critic (**Figure** **1**). The generator receives stochastically random noise and produces artificial data samples. These are passed to the critic along with real data samples. The critic evaluates these samples for their probability and computes the Wasserstein distance (also known as ‘earth mover's distance’) between the real and generated data distributions. The negative of the Wasserstein distance estimate is the loss function for the critic.

To correctly compute the Wasserstein distance, the critic must be a 1‐Lipschitz function, meaning that the gradients of the critic with respect to its inputs must have a norm of at most 1. To enforce this constraint, the gradient penalty adds a regularization term to the loss function that penalizes the critic if the norm of its gradients deviates from 1. This gradient penalty results in more stable training and better convergence properties by effectively enforcing the Lipschitz constraint [9, 10].

The critic's estimates of the artificial samples are used as a loss function for the generator, which implicitly learns to produce data that minimize the Wasserstein distance to the real data distribution. Over time, the generator improves its output, eventually creating new data that are indistinguishable from real data, though not identical to any specific real data observation.

As the critic's feedback is crucial for training the generator, the critic must sufficiently approximate the Wasserstein distance. If the critic is untrained, the estimated distance is noisy or wrong. Consequentially, the generator is optimized based on these unreliable gradients from the critic, which might lead to unstable training. Therefore, we train and update the critic five times more often than the generator, as proposed in the literature [9].

### 2.2 Original Patients

We created a data set of 20 original patients, with a random age range of 18 to 65 years, comprising 10 female and 10 male patients. The weights of females and males were normally distributed samples with a mean of 64 kg and 85 kg, respectively, and a standard deviation of 10 kg [11, 12]. The heights of females and males were normally distributed samples with a mean of 164 cm and 179 cm, and a standard deviation of 7 cm [11, 12]. We used these patients to simulate the blood concentration after extravascular administration of 300 mg of a hypothetical drug with first‐order absorption, a one‐compartment distribution, and linear elimination. Weight is a covariate influencing volume of distribution with a coefficient of 0.75. The ground truth of the population parameters is shown in Table 1. Blood concentrations were simulated at 0.5, 1, 1.5, 2, 4, 6, 8, and 12 h after administration. We censored concentrations below 0.5 mg/L as being below limit of quantification (BLQ). Ground truth and parameter estimates from the different experimental setups are shown in Table 1. Additional information, that is R code base for generation of the original patients, simulation of concentration curves based on ground truth, transformed original data set for WGAN‐GP training, and transformed original data set for PopPK modeling are provided at https://github.com/cptbern/WGAN_PopPK.

**TABLE 1:** Population pharmacokinetic parameters: Ground truth and results of PopPK modeling with different data sets.

Parameter	Ground truth	Parameter estimates [Bootstrap analysis: Median, 95% CI]				
		Original	Artificial	Combined‐low	Combined‐equal	Combined‐high
**Fixed effects**						
Absorption rate constant (ka_pop_) [h^−1^]	0.5	0.53 [0.53, 0.3–0.65]	0.55 [0.54, 0.35–0.65]	0.48 [0.49, 0.3–0.61]	0.55 [0.56, 0.33–0.65]	0.52 [0.54, 0.36–0.65]
Volume of distribution V_pop_ [L]	14.0	16.26 [15.88, 9.04–20.54]	16.86 [16.51, 10.92–19.75]	14.81 [14.36, 9.16–19.8]	16.45 [16.49, 9.66–19.55]	15.92 [16.33, 11.53–19.25]
Clearance Cl_pop_ [L/h]	5.0	5.01 [5.05, 4.65–5.48]	5.02 [5.05, 4.75–5.28]	5.11 [5.08, 4.75–5.41]	5.02 [4.99, 4.71–5.35]	5.06 [5.05, 4.78–5.34]
Coefficient of log_weight_ on V_pop_	0.75	0.65 [0.71, −0.2‐1.71]	0.73 [0.77, 0.065–1.29]	0.82 [0.95, 0.18–1.89]	0.79 [0.87, 0.16–1.75]	0.85 [0.72, 0.10–1.34]
**Inter‐individual variability (IIV)**						
ka_IIV_	0.2	0.15 [0.2, 0.057–0.36]	0.13 [0.21, 0.079–0.35]	0.11 [0.21, 0.067–0.41]	0.19 [0.16, 0.075–0.35]	0.28 [0.24, 0.10–0.38]
V_IIV_	0.3	0.31 [0.24, 0.073–0.38]	0.31 [0.26, 0.11–0.34]	0.38 [0.29, 0.066–0.43]	0.32 [0.29, 0.1–0.38]	0.24 [0.27, 0.10–0.35]
Cl_IIV_	0.2	0.16 [0.16, 0.1–0.2]	0.16 [0.16, 0.13–0.19]	0.18 [0.18, 0.12–0.22]	0.18 [0.18, 0.14–0.22]	0.18 [0.17, 0.14–0.2]
**Residual error**						
Additive error (a) [mg/L]	0.2	0.34 [0.33, 0.15–0.51]	0.51 [0.5, 0.34–0.65]	0.43 [0.42, 0.25–0.58]	0.39 [0.39, 0.23–0.54]	0.49 [0.5, 0.34–0.63]
Proportional error (b)	0.1	0.077 [0.076, 0.044–0.11]	0.049 [0.05, 0.024–0.076]	0.066 [0.065, 0.047–0.1]	0.07 [0.072, 0.049–0.1]	0.052 [0.051, 0.029–0.077]

*Note:* Combined‐low: 20 original and 10 artificial patients; combined‐equal: 20 original and 20 artificial patients; combined‐high: 20 original and 40 artificial patients.

### 2.3 Artificial Patients With WGAN‐GP

Artificial patients were generated by a WGAN‐GP [9, 10]. All patient characteristics of the original data set, including the estimated blood concentrations, were scaled to [−1, 1], and the categorical variable sex was one‐hot encoded (separating the sex column into two columns “male” and “female” with binary encoding). Although this is an unorthodox way of handling binary variables in pharmacometrics, it is common in ML and easily interconverted.

The critic and the generator networks consisted of two hidden layers. Additionally, the critic had two dropout layers. As we applied gradient penalty, we did not use any batch normalization layer in the generator [10]. We used a leaky rectifier linear unit (ReLu) for the activation function, except for the output layer, where we used a linear activation function. As an optimizer, we used an optimistic ADAM (OADAM), which is a variant of ADAM that adds an *optimistic* term suitable for adversarial training [13]. The learning rate was set to 1 × 10^−4^ for the generator and critic. The critic was trained five times more often than the generator. Overall, the WGAN was trained over 150′000 epochs.

As the generator creates artificial patients at random, creation of a small subset might lead to a sample which does not cover the problem space. However, adding a skewed sample to the original patients might move the parameter estimation away from the population estimates. Therefore, we decided it is vital to obtain a subsample which reflects the whole problem space. To obtain the most diverse subset of samples from the fully trained generator for PopPK modeling, we created 200 artificial data sets (factor of 10). We then calculated the pairwise Euclidean distances between them and heuristically selected samples iteratively by maximizing the minimum distance to already selected samples, ensuring that selected samples are as different from each other as possible.

More details on the WGAN‐GP, that is, Julia code base including parameter definitions and architecture of the WGAN‐GP, generated artificial patients, and heuristic artificial patient selection are provided at https://github.com/cptbern/WGAN_PopPK.

### 2.4 PopPK Model Building

As the original data set was scaled from [−1, 1] for training of the WGAN‐GP, the results of the artificial patients were also within this range. Therefore, we re‐converted these values using the conversion factors of the original patients before proceeding to PopPK modeling. In addition, we merged the one‐hot encoded categorical variable into a single column using one‐cold encoding, complying with pharmacometric practice and what is most easily processed in standard software tools. After conversion, we censored all values below 0.5 to mimic the exclusion of BLQ values.

We created and compared five data sets with different shares of original and artificial patients; details are provided in Table 2.

**TABLE 2:** Population data sets.

Name	Amount original patients	Amount artificial patients	Total
Original	20	0	20
Artificial	0	60	60
Combined‐low	20	10	30
Combined‐equal	20	20	40
Combined‐high	20	40	60

We fitted the data sets to PopPK models and obtained the parameter estimates for the fixed and random effects. We then conducted a non‐parametric bootstrap (*n* = 200) to validate each model. We compared the obtained pharmacokinetic parameter estimates to the ground truth and with each other.

### 2.5 Statistical Evaluation

In addition to the indirect, model‐based comparison of the concentration curves, we also wanted to compare the data distributions of the original and artificial patients directly. This shall ensure that there are no significant differences in the data distribution of the original and artificial patients, thus indirectly providing information of the performance of the generator.

However, as the concentration changes over time (absorption phase, peak and elimination phase), the overall distribution is not stationary, but inherently time‐dependent. If we just compared the data distribution of each time point separately, we would only analyze pointwise similarity, but ignore the underlying structure, that is the changes of the drug concentration over time. Consequentially, the curves from both groups should be considered as samples from the same multivariate distribution. Therefore, we decided to use the non‐parametric statistical test Maximum Mean Discrepancy (MMD) [14]. The MMD quantifies the difference of two sample sets by comparing their means in a high‐dimensional feature space induced by a kernel function. If the two distributions are the same, the mean embeddings of their samples in this feature space will be close, resulting in small MMD values. We used a Gaussian RBF kernel and a sigma of 0.1.

### 2.6 Software

Data wrangling and statistics were performed in GNU R (version 4.2.2, R Foundation for Statistical Computing, http://www.R‐project.org, Vienna, Austria), as well as simulation of concentration profiles using the package RsSimulx: R speaks ‘Simulx’ (version 1.0.1, [15]). Training of WGAN‐GP were performed in Julia Language (version 1.10.3, [16]) using the Flux package (version 0.14.15). Building of PopPK models and bootstrap analysis were done in Monolix (version 2024R1, Antony, France: Lixoft SAS, 2024).

## 3 Results

### 3.1 PopPK Models

In the original data set, the estimates of the central volume of distribution (Vd) were higher and the coefficient of weight on Vd was lower than the ground truth. The absorption rate constant (ka) and clearance (Cl) were reasonably well estimated (Table 1). During model fitting, residual error was considered to be additive, leading to a larger additive and a smaller proportional error compared to ground truth. All point estimates are within the confidence interval of the bootstrap analysis. However, the coefficient of weight on Vd has a substantial confidence interval which also includes zero.

The models using the artificial and combined data sets yield comparable results to those obtained with the original data set, with minor deviations in the point estimates. Noteworthy here is that the influence of weight on Vd is always positive in the bootstrap analysis. Additionally, the confidence intervals are generally narrower compared to the original data set.

Visual Predictive Checks (VPC) of all data sets are provided in the supporting information (Figure S1).

### 3.2 Statistical Evaluation

**FIGURE 2:**
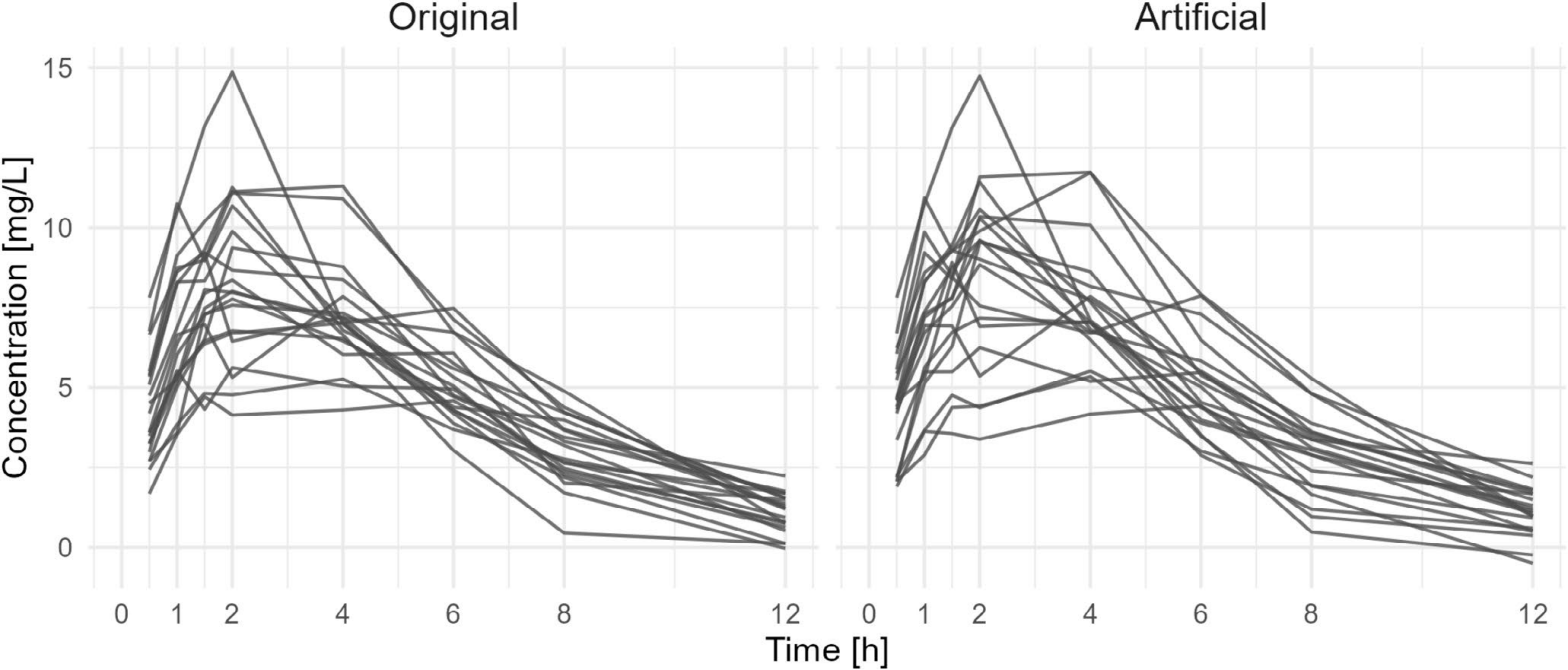
Comparison of original and artificial concentration curves.

Visually, the concentration curves of the original and artificial patients look comparable (**Figure** **2**). However, to determine if the concentration curves could be from the same population, we used a non‐parametric test (MMD) that compares the probability distributions between original and artificial patients. Due to the resulting low MMD^2^ statistic value (MMD^2^ = 0.048) and the high *p*‐value (*p* = 0.995), we accept the null hypothesis that the original and artificial patients are from the same data distribution. Changes in sigma did not result in any changes to the overall.

## 4 Discussion

In this study, we used an implementation of generative artificial intelligence, specifically WGAN‐GP, for generating artificial patient profiles. For this purpose, we provided the WGAN‐GP with original patient information, specifically the covariates sex, age, height, weight, and dose, along with drug concentrations at eight time points. We specifically used a small data set of only 20 patients to explore and push the boundaries of model performance. We applied three complementary validation strategies by (i) visually comparing concentration–time profiles, (ii) assessing distributional similarity using MMD, and (iii) comparing parameter point estimates of the PopPK model based on the different data sets. This multi‐level validation framework was intended to reduce the risk of overinterpreting any single metric and to provide converging evidence that the WGAN captured relevant structural properties of the data. Even with this limited information, the WGAN‐GP was able to produce artificial patients with comparable data distributions to the original data set. This did not only include constant patient parameters (age, weight, height) but also time‐varying information (blood concentration curves). Therefore, we can conclude that the WGAN‐GP did converge and no mode collapse occurred.

The concentration‐time profiles of original and artificial patients were comparable. The MMD was not able to detect a significant difference between the original and artificial patients. The point estimates of the subsequently PopPK models built solely with artificial patients, and combinations with original patients, were comparable to those built only from original patients (observed data). The WGAN‐GP was able to map the covariate influence on the PK parameters and even accentuate it. In the model with the original data set, the confidence interval for the weight coefficient included zero, suggesting that there might not be enough evidence of an influence. The addition of the artificial patients led to the exclusion of zero from the confidence interval. Furthermore, the overall narrower confidence intervals suggest more precise point estimates.

It is interesting to note that we did not provide the concentration measurements as time series, but merely as independent points within the data set. Still, the WGAN‐GP was able to pick up on the connection between these points without being provided further information. This is further evidence for the power of generative AI in this application.

This work was designed as a proof‐of‐concept study, and as such has limitations, which are important to bear in mind. We assumed a rich data sampling strategy with no missing information and absolute precision in recording of time and observations. In clinical practice, this will likely be the case. Furthermore, while the vast majority of runs did converge, single training runs did not, even with the same parameters. So, after training the WGAN‐GP, the output needs to be monitored. In our case, non‐converging runs were easily identifiable by plotting the concentration curves, which were erratic and not representative of the original patients in non‐converging runs. In contrast, even though not observed during this study, the training of the WGAN‐GP might result in a mode collapse, where the generator only creates samples based on few or single instances of the training data set.

Additionally, we used a non‐parametric test (MMD) to compare the probability distributions. For the ultimately used artificial data samples, the non‐parametric test indicates that the distribution between the original and artificial data are comparable. However, we only had 20 samples per group, which limit the ability to detect differences.

Generated data sets cannot truly introduce independent information, but are essentially an interpolation of the original data. The narrowing of the confidence intervals is solely driven by the increase in sample size (a statistical effect), and not by new information. While the WGAN‐GP might amplify real trends in the original data, it also amplifies biased trends inherent to numerical identifiability issues. While generative AI research commonly uses artificial data to augment the data the generative model was trained on, it leaves the risk of circularity and information leakage. This issue could be mitigated by training the generative AI model on one subset of data with the augmentation step done on a separate subset. However, as we intentionally used small data sets (20 original patients) in this proof‐of‐concept study, we decided against that approach.

Considering all the limitations, we currently consider this approach solely suitable for exploratory analysis and not as a replacement for confirmatory clinical trial analysis based on real patient data.

In conclusion, generation of artificial patients using WGAN‐GP for augmentation of pharmacometrics data sets appears feasible and merits further study. Specific areas of interest could be more complex structural models, sparse/irregular sampling, missing data, or evaluation on its use on real‐world data sets.

## Author Contributions

**Verena Schöning:** conceptualization, methodology, data curation, writing – original draft, writing – review and editing, visualization, formal analysis, investigation, software. **Felix Hammann:** conceptualization, methodology, software, supervision, visualization, writing – review and editing.

## Funding

The authors have nothing to report.

## Ethics Statement

The authors have nothing to report.

## Conflicts of Interest

The authors declare no conflicts of interest.

## Supporting information


**Figure S1:** Visual Predictive Checks (VPC) of all analyzed data sets.

## Data Availability

The data that support the findings of this study are openly available in Github at https://github.com/cptbern/WGAN_PopPK.

## References

[1] FDA , “Guidance for Industry: Population Pharmacokinetics,” in , ed. Services USDoHaH, (CDER) CfDEaR, (CBER) CfBEaR (1999).

[2] A. Janssen , F. C. Bennis , and R. A. A. Mathôt , “Adoption of Machine Learning in Pharmacometrics: An Overview of Recent Implementations and Their Considerations,” Pharmaceutics 14, no. 9 (2022): 1814. 36145562 10.3390/pharmaceutics14091814 PMC9502080

[3] Y. Jiang , A. García‐Durán , I. B. Losada , P. Girard , and N. Terranova , “Generative Models for Synthetic Data Generation: Application to Pharmacokinetic/Pharmacodynamic Data,” Journal of Pharmacokinetics and Pharmacodynamics 51, no. 6 (2024): 877–885, 10.1007/s10928-024-09935-6. 39192091

[4] A. Janssen , L. Smalbil , F. C. Bennis , et al., “A Generative and Causal Pharmacokinetic Model for Factor VIII in Hemophilia A: A Machine Learning Framework for Continuous Model Refinement,” Clinical Pharmacology and Therapeutics 115, no. 4 (2024): 881–889, 10.1002/cpt.3203. 38372445

[5] A. Nikolopoulos and V. D. Karalis , “Implementation of a Generative AI Algorithm for Virtually Increasing the Sample Size of Clinical Studies,” Applied Sciences 14, no. 11 (2024): 4570.

[6] I. J. Goodfellow , J. Pouget‐Abadie , M. Mirza , et al., “Generative adversarial nets Proceedings of the 27th International Conference on Neural Information Processing Systems,” (2014).

[7] I. Goodfellow , J. Pouget‐Abadie , M. Mirza , et al., “Generative Adversarial Networks,” Communications of the ACM 63, no. 11 (2020): 139–144, 10.1145/3422622.

[8] S. J. Nightingale and H. Farid , “AI‐Synthesized Faces Are Indistinguishable From Real Faces and More Trustworthy,” Proceedings of the National Academy of Sciences 119, no. 8 (2022): e2120481119, 10.1073/pnas.2120481119. PMC8872790 35165187

[9] M. Arjovsky , S. Chintala , and L. Bottou , “Wasserstein Generative Adversarial Networks,” in Proceedings of the 34th International Conference on Machine Learning, Proceedings of Machine Learning Research, eds. P. Doina and T. Yee Whye , 70 (PMLR, 2017), 214–223.

[10] I. Gulrajani , F. Ahmed , M. Arjovsky , V. Dumoulin , and A. C. Courville , “Improved Training of Wasserstein Gans,” Advances in Neural Information Processing Systems 30 (2017): 1–11.

[11] “Worlddata.info: Average sizes of men and women,” (2022), https://www.worlddata.info/average‐bodyheight.php.

[12] K. Staub , F. Rühli , U. Woitek , and C. Pfister , “The Average Height of 18‐ and 19‐Year‐Old Conscripts (*N*=458,322) in Switzerland From 1992 to 2009, and the Secular Height Trend Since 1878,” Swiss Medical Weekly 141 (2011): w13238, 10.4414/smw.2011.13238. 21805409

[13] C. Daskalakis , A. Ilyas , V. Syrgkanis , and H. Zeng , “Training GANs With Optimism,” arXiv (2017) abs/1711.00141.

[14] A. Gretton , K. M. Borgwardt , M. J. Rasch , B. Schölkopf , and A. Smola , “A Kernel Two‐Sample Test,” Journal of Machine Learning Research 13, no. 25 (2012): 723–773.

[15] C. Pinaud and J. Chauvin , “RsSimulx: R Speaks'Simulx',” (2021).

[16] J. Bezanson , A. Edelman , S. Karpinski , and V. B. Shah , “Julia: A Fresh Approach to Numerical Computing,” SIAM Review 59, no. 1 (2017): 65–98, 10.1137/141000671.

