# Supplementary material for: Improving Population Pharmacokinetic Modelling with Artificial Patients using Generative Artificial Intelligence
Source: Pharmacol Res Perspect. 2026 Apr 3;14(2):e70241. doi: 10.1002/prp2.70241 (PMC13052321; doi:10.1002/prp2.70241)
Supplement: Supplementary file 1 — Figure S1: Visual Predictive Checks (VPC) of all analyzed data sets. [file PRP2-14-e70241-s001.pdf]

Supplementary Information:

Improving Population Pharmacokinetic Modelling with Artificial Patients using Generative Artificial Intelligence

Verena Schöning, Felix Hammann

Figure S1: Visual Predictive Checks (VPC) of all analysed datasets 2

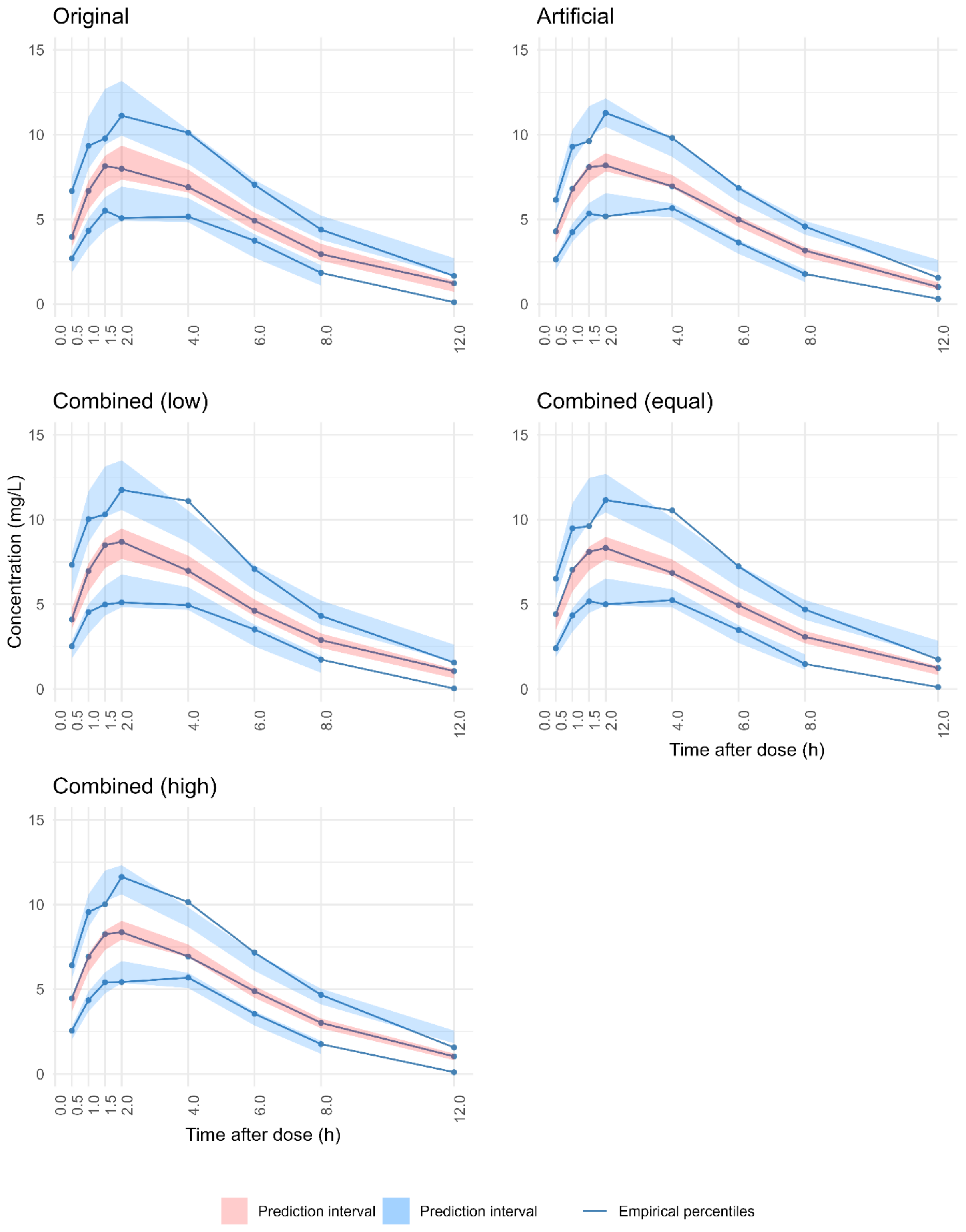

Figure S1: Visual Predictive Checks (VPC) of all analysed datasets
